# NK Cell Influence on the Outcome of Primary Epstein–Barr Virus Infection

**DOI:** 10.3389/fimmu.2016.00323

**Published:** 2016-08-29

**Authors:** Obinna Chijioke, Vanessa Landtwing, Christian Münz

**Affiliations:** ^1^Institute of Surgical Pathology, University Hospital Zürich, Zürich, Switzerland; ^2^Viral Immunobiology, Institute of Experimental Immunology, University of Zürich, Zürich, Switzerland

**Keywords:** lytic EBV infection, NKG2D, DNAM-1, infectious mononucleosis, humanized mice

## Abstract

The herpesvirus Epstein–Barr virus (EBV) was discovered as the first human candidate tumor virus in Burkitt’s lymphoma more than 50 years ago. Despite its strong growth transforming capacity, more than 90% of the human adult population carries this virus asymptomatically under near perfect immune control. The mode of primary EBV infection is in part responsible for EBV-associated diseases, including Hodgkin’s lymphoma. It is, therefore, important to understand which circumstances lead to symptomatic primary EBV infection, called infectious mononucleosis (IM). Innate immune control of lytic viral replication by early-differentiated natural killer (NK) cells was found to attenuate IM symptoms and continuous loss of the respective NK cell subset during the first decade of life might predispose for IM during adolescence. In this review, we discuss the evidence that NK cells are involved in the immune control of EBV, mechanisms by which they might detect and control lytic EBV replication, and compare NK cell subpopulations that expand during different human herpesvirus infections.

## Epstein–Barr Virus Infection and Predisposing Factors for EBV Disease

The herpesvirus Epstein–Barr virus (EBV) was discovered in 1964 by electron microscopy in Burkitt’s lymphoma, the most common childhood tumor in sub-Saharan Africa (1). It is arguably the most potent human tumor virus, because it readily transforms primary human B cells into immortalized lymphoblastoid cell lines (LCLs) in culture (2). This strong growth transforming capacity is due to the latent EBV proteins, six nuclear antigens (EBNAs) and two latent membrane proteins (LMPs), which are expressed as the default infection program in B cells (3). Lytic EBV replication occurs in LCLs only at low levels and triggers the expression of around 80 gene products under the guidance of the immediate early lytic transactivator BZLF-1 for the production of infectious DNA virus particles (4). In addition to Burkitt’s lymphoma, EBV is associated with numerous malignancies, mostly of B and epithelial cell origin, such as Hodgkin’s lymphoma and nasopharyngeal carcinoma (3). Despite this strong growth transforming capacity, EBV is carried by more than 90% of the human adult population as an asymptomatic persistent infection.

**Figure 1:**
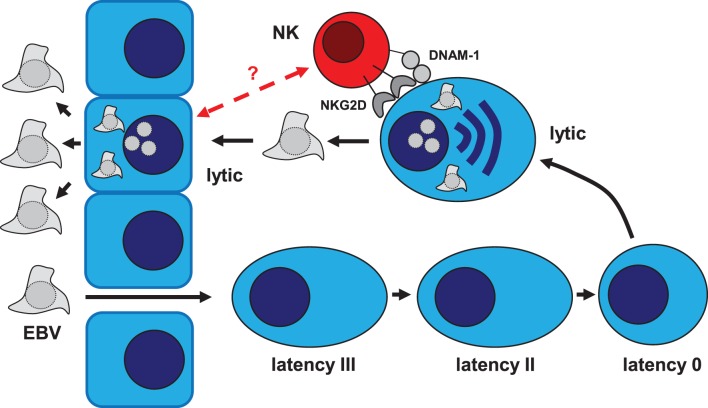
**Role of NK cells in the immune control of the EBV life cycle**. Epstein–Barr virus (EBV) is transmitted via saliva exchange and infects submucosal B cells. In infected naïve B cells, the latency III EBV program can be found (EBNA1, 2, 3A–C, LP, and LMP1 and 2). Activation via EBV infection drives infected B cells into differentiation. In resulting germinal center B cells, latency II EBV infection can be found (EBNA1, LMP1 and 2). These EBV proteins allow EBV-infected B cells to survive and enter the memory B cell pool. In memory B cells, all EBV proteins expression is switched off (latency 0). Upon B cell receptor cross-linking, the lytic EBV cycle is activated due to plasma cell differentiation, which allows epithelial cell infection for further amplification of infectious virus before shedding into saliva. NK cells target lytically EBV replicating cells via their activating NKG2D and DNAM-1 receptors. It remains unclear if also lytically EBV replicating epithelial cells can be recognized by NK cells.

Epstein–Barr virus infection remains asymptomatic in most persistently infected individuals despite transforming latent EBV protein expression (5). In healthy EBV carriers, the expression of all six EBNAs and the two LMPs can be found in naïve B cells of secondary lymphoid organs like the tonsils (6) (Figure 1). In germinal center B cells, only the subset of viral proteins that is also present in Hodgkin’s lymphoma is expressed (EBNA1, LMP1 and 2). Finally, in homeostatically proliferating memory B cells, the latency pattern of Burkitt’s lymphoma is present with EBNA1 as the only expressed protein (7). Reactivation from this persistent EBV reservoir of memory B cells into lytic replication seems to occur after B cell activation and plasma cell differentiation (8). These findings, however, indicated that healthy EBV carriers are continuously challenged with transforming latent EBV expression programs, which could result in tumor formation without immune control.

Indeed, immune suppression after organ transplantation or due to human immunodeficiency virus (HIV) co-infection causes the occurrence of EBV-associated malignancies, such as post-transplant lymphoproliferative disease (PTLD) and immunoblastic lymphoma (3). Some of these lymphoproliferations can be treated by the adoptive transfer of EBV-specific T cell lines (9). Moreover, some individuals remain EBV seronegative despite carrying the virus, and seem to control persistent EBV infection entirely by cell-mediated immunity (10). Thus, cell-mediated immunity by T cells seems to be sufficient to control persistent EBV infection (11). In addition to direct immune suppression of cell-mediated immunity, the conditions under which this immune response is primed, seems to be decisive for an asymptomatic immune control of EBV infection. Indeed, if primary infection with EBV is delayed into adulthood, the virus is more frequently acquired with symptomatic primary infection, called infectious mononucleosis (IM) (12). This immunopathology by massive CD8^+^ T cell expansion and activation seems to result from an uncontrolled lytic EBV replication, because most of the expanding CD8^+^ T cells are directed against lytic EBV antigens (13). This massive lymphocytosis seems to transiently compromise EBV-specific immune control with an increased susceptibility to some EBV-associated malignancies, such as Hodgkin’s lymphoma up to 5–10 years after IM (14). In this review, we discuss the innate arm of cell-mediated immune control of EBV, which could explain the different outcomes of primary infection with this tumor virus and might be required to limit initial viral titers so that long-lasting adaptive cell-mediated immune control can be efficiently primed.

## Primary Immunodeficiencies That Affect NK Cell Function and Predispose for EBV Disease

Evidence that loss of cytotoxic cell-mediated immune control predisposes for EBV-associated diseases comes from primary immunodeficiencies that sensitize for EBV-associated malignancies (15, 16). A subset of these affect, in addition to T cell responses, natural killer (NK) cell responses and hint toward an important function of cell-mediated innate immunity in EBV-specific immune control. The underlying genetic lesions affect gene products that are involved in NK cell differentiation, stimulation, and cytotoxic effector function.

Natural killer cell differentiation is disrupted by mutations in GATA-binding protein 2 (GATA2) and minichromosome maintenance complex component 4 (MCM4). Accordingly, a GATA2 mutation was identified later in the first indicator patient with susceptibility to herpesvirus induced diseases (17, 18). GATA2 is a hematopoietic transcription factor that is required for the development of several immune cell lineages, including B cells, CD4^+^ T cells, dendritic cells, neutrophils, and monocytes in addition to NK cells (19). With respect to EBV-associated diseases, patients with GATA2 mutations have been diagnosed with chronic active EBV infection (CAEBV) and virus-positive smooth muscle tumors (20, 21). In contrast to this multilineage deficiency in patients with GATA2 mutations, partial deficiency of the DNA helicase MCM4 blocked differentiation of the human CD56^dim^ NK cell subset, while other hematopoietic lineages seemed to be unaffected (22). One of the afflicted patients suffered from an EBV-associated lymphoma (23). Thus, compromised NK cell differentiation is associated with uncontrolled EBV infection.

In addition to NK cell differentiation, some mutations that impact NK cell stimulation are associated with EBV disease. These include deficiencies in SLAM-associated protein (SAP) of X-linked lymphoproliferative disease type 1 (XLP1), in magnesium transporter 1 (MAGT1) of X-linked immunodeficiency with magnesium defect, EBV infection, and neoplasia (XMEN), in CD27, in phosphatidylinositol 3 kinase (PI3K) 110δ and in FcγR3A (CD16). XLP1, also known as Duncan’s disease, primarily manifests in boys (24). Primary infection with EBV often leads to fatal IM in the affected patients, if they cannot be identified early enough and treated with bone marrow transplantation (25). The underlying mutations in SAP were identified in 1998 (26–28) and affect the adaptor protein of SLAM receptors that mediate their co-activating function in T and NK cells. Two of these SLAM receptors, 2B4 and NTB-A, increase NK cell cytotoxicity (29, 30), but XLP1-associated SAP mutations might primarily compromise EBV-specific CD8^+^ T cell immune control (31–33). Furthermore, deficiency in the magnesium transporter MAGT1 results in diminished free magnesium levels within cells, which is associated with downregulation of the activating NKG2D receptor on cytotoxic lymphocytes, T, and NK cells (34). Supplementation of magnesium results in decreased EBV loads in the affected XMEN patients. Another activating co-receptor on T and NK cells is CD27. Mutations in this CD70 engaging co-receptor predispose for EBV-associated lymphoproliferations (35, 36). Also, loss-of-function mutations in the signaling molecule PI3K 110δ of activating receptors are associated with persistent EBV viremia (37). Finally, the activating FcγR on NK cells, CD16, seems to be required for EBV-specific immune control. Mutations in CD16 were reported to be associated with persisting IM symptoms (38, 39). These primary immunodeficiencies identify 2B4, NKG2D, CD27, and FcγR as important receptors in EBV-specific cell-mediated immune control.

Apart from these activating receptors, the cytotoxic effector machinery also seems to be important in EBV-specific immune control. Accordingly, mutations in perforin, Munc13-4, and Munc18-2 have been identified in patients with EBV-associated diseases. Mutations in perforin are responsible for type 2 familial hemophagocytic lymphohistiocytosis (FHL2). Persistent IM has been described in one FHL2 patient (40). Munc18-2 and 13-4 mediate docking and activation of syntaxin 11 for cytotoxic granule fusion with the cell membrane, respectively. Mutations in these two components of the cytotoxic machinery were found in patients with CAEBV (41). These genetic lesions point toward a role of cytotoxic lymphocytes in EBV-specific immune control. Primarily, prolonged IM resulting in CAEBV seems to be associated with primary immunodeficiencies that affect NK cell function.

## NK Cell Expansion During Primary EBV Infection

**Figure 2:**
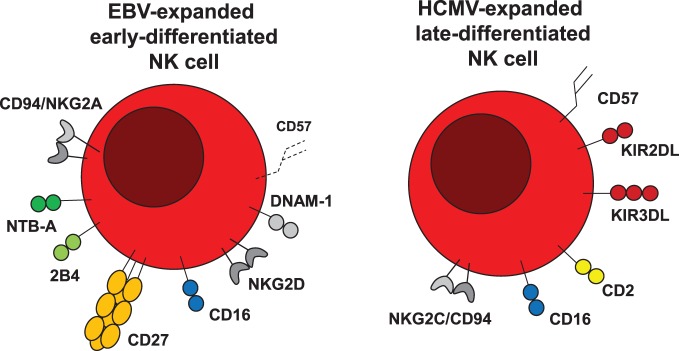
**EBV and HCMV expand different NK cell populations**. Epstein–Barr virus (EBV) expands early-differentiated NKG2A/CD94, NTB-A, 2B4, CD27, CD16, NKG2D, and DNAM-1-positive NK cells, which after expansion upregulate the senescence marker CD57. Human cytomegalovirus (HCMV) in contrast expands late-differentiated CD94/NKG2C, CD16, CD2, KIR, and CD57-positive NK cells.

Natural killer cell expansion during primary EBV infection has first been reported in a study by Tomkinson et al. (42), in which peripheral NK cells (identified as CD16^+^ lymphocytes) were described to be significantly increased in both frequency (1.5-fold) and absolute number (4-fold) in – by these measures – a similar manner to CD8^+^ T cells in a cohort of IM patients. However, since the authors had to use a strategy for gating lymphocytes that included activated and, thus, blasted cells, CD16^+^ monocytes could not be excluded from the analysis and might account for some of the quantitative changes ascribed to the NK cell compartment. Still, threefold to sixfold increases in the number of bulk NK cells in IM patients were found by other groups as well (43, 44) and these increases were found to be inversely correlated with viral load in blood (43). Likewise, higher NK cell counts tended to be associated with less severe disease (43). On the contrary, a large and, notably, prospective study of primary EBV infection (45), while also reporting expansions of NK cells during the acute phase, positively correlated NK cell numbers with blood viral load and also positively correlated blood viral load with disease severity (45). Similarly, the increase in NK cells in IM patients was related to greater disease severity by another group, although the small number of subjects in that study precluded statistical significance (44). A study by Azzi et al. (46) detailed the phenotype of NK cells during IM and convalescence in pediatric patients and demonstrated the lack of influence of primary EBV infection on the expression of killer cell immunoglobulin-like receptors (KIRs) but instead noted an up to fivefold expansion of an early-differentiated NK cell subset (Figure 2). This accumulated NKG2A^+^KIR^−^CD57^−^ NK cell subset was the only identifiable subset within the NK cell compartment that proliferated in the acute phase and importantly, this proliferating early-differentiated NK cell subset also correlated with viral load in PBMCs (46). Although overall NK cell numbers and frequencies contract early after the onset of symptoms (43–46), these early-differentiated NK cells remain elevated in frequency up to 6 months after the acute symptomatic phase (46–48), but over time accumulate signs of differentiation (46, 47). Asymptomatic primary EBV infection is mostly found in young children (49, 50) compared to a symptomatic outcome, i.e., IM, in up to 75% of cases of primary EBV infection in adolescents (45). While asymptomatic infection was associated with high viral load, phenotype, and frequencies of antigen-specific CD8^+^ T cells similar to IM, the massive expansion of CD8^+^ T cell numbers typically seen in IM was absent (49). It might be speculated that the confinement of CD8^+^ T cell expansion is exerted by the EBV-responsive early-differentiated NKG2A^+^KIR^−^ NK cell subset, especially since this subset is highest in both frequency and numbers in newborns and young children but decreases with age (46). Whether the loss of early-differentiated NK cells during adolescence is associated with a specific molecular imprint that affects NK cell homeostasis, e.g., the result of changes in the expression of transcription factors, has not yet been explored in the current literature. One explanation for such an age-dependent effect, however, is an increased burden and accumulation of various infectious challenges with advancing years that can likely be expected to have an impact on the differentiation of NK cells. One of these challenges, namely infection with the human cytomegalovirus (HCMV), that seems to drive NK cell differentiation via IL-12 and IL-15 production, is discussed below. Thus, dynamics within the NK cell compartment over time might in part explain the age-dependent occurrence of symptomatic primary EBV infection.

## NK Cell Reactivity Against Lytic EBV Infection

Indeed, the trigger of peripheral NK cell accumulation in primary EBV infection does not seem to be caused by the inflammatory status of IM itself, e.g., increased levels of pro-inflammatory cytokines, since patients with equally inflammatory conditions but lacking evidence of EBV seroconversion do not show any expansions in their NK cell compartment (46). Instead, there is evidence that the state of the infectious cycle of EBV, either latent or lytic, drives the expansion of NK cells during infection, specifically changes that are inherent to lytic replication. In mice with reconstituted human immune system components (HIS mice), NK cell expansion only occurs during infection with wild-type EBV, but not with recombinant EBV engineered to only establish latent infection (EBV BZLF-1 knockout or BZ1KO EBV) (51). Furthermore, proliferation of NKG2A^+^KIR^−^ NK cells was only seen after *in vitro* infection of cord blood with wild-type EBV but not with BZ1KO EBV (46). It is, therefore, conceivable that the expansion of the cytotoxic lymphocyte populations, namely NK and CD8^+^ T cells, during EBV infection is driven by the amount of available antigen (45, 46, 51), since the expansion of total CD8^+^ T cell and NK cell numbers as well as viral load correlate (45). Actually, lytic replication might not only be responsible for the expansion of the early-differentiated NK cell subset, but seems to also be a target of NK cells itself (Figure 1). In EBV-infected HIS mice, NK cells protect from high viral load, elevated cytokine levels, splenomegaly, weight loss, and occurrence of lymphoproliferative tumors, as well as limit the expansion of CD8^+^ T cells (51). Most of the protective effects of NK cells are lost in HIS mice only latently infected with EBV, but regained when these mice are infected with a recombinant virus reverted to allow for lytic replication (51). Also, in EBV-infected HIS mice depleted of NK cells, there is an increased abundance of lytic proteins and cell-free viral DNA indicative of ongoing uncontrolled lytic replication (51). *In vitro*, NK cells respond to and kill an EBV-positive B cell line more efficiently when these cells are in the lytic as compared to the latent phase of infection (51–53), in particular NK cells with the NKG2A^+^KIR^−^ phenotype (46). The preferential killing of lytic cells was sensitive to blocking of CD112 and ULBP-1, ligands of the activating NK cell receptors DNAM-1 and NKG2D, respectively (52) as well as directly blocking DNAM-1 (53). Therefore, the identification of activating receptors or combinations thereof crucial in NK cell-mediated protection *in vivo* holds promise to further our understanding of the intricate interplay between EBV with the host’s immune system and HIS mice might constitute a feasible model to answer such questions (51).

## Differences Between EBV-Driven NK Cell Expansion and Other Human Herpesvirus Infections

In contrast to EBV infection, other herpesviruses either do not change the NK cell composition, such as recurrent α-herpesvirus infection by herpes simplex virus 2 (HSV2) (54), or expand terminally differentiated NKG2C^+^KIR^+^CD16^+^ NK cells, such as the β-herpesvirus HCMV (55–57) or the γ-herpesvirus Kaposi sarcoma-associated herpesvirus (KSHV) in HIV-infected individuals (58). Accumulation of terminally differentiated NK cells is primarily connected to HCMV infection (Figure 2) and it has been argued that in other viral infections, for which such terminal NK cell differentiation can be observed, such as with Hantavirus (59), Chikungunya virus (60), HIV (61), and hepatitis virus (62), mainly HCMV-positive individuals are affected by this alteration in NK cell repertoire composition (63–65).

This HCMV-driven terminal NK cell expansion has been linked to NK cell stimulation by cells that produce the NKG2C ligand HLA-E plus the NK cell proliferation stimulating cytokine IL-15 on their surface (59, 66). Expansion of NKG2C-positive NK cells could be obtained with HCMV infected fibroblasts plus IL-15 (67) and bystander monocytes were able to provide NK cell stimulating cytokines, including IL-12 (68). However, HCMV-infected individuals with NKG2C deficiency also develop NK cell populations that more vigorously secrete IFN-γ upon stimulation, the so-called adaptive NK cell populations (69), and the NKG2C genotype does not affect the outcome of congenital HCMV infection (57). Therefore, HLA-E-mediated NK cell stimulation might not be essential for the expansion and anti-viral function of NKG2C-positive NK cell populations, but IL-15 and IL-12 might be more important (65). Accordingly, one patient with IL-12Rβ1 deficiency did not carry adaptive NK cell populations (70). Therefore, cytokines might be one of the main drivers of adaptive NK cell expansion, as originally proposed in mice (71). These adaptive NK cells are terminally differentiated NKG2C-positive NK cells during HCMV infection, while for the early-differentiated NK cells that expand and persist for 6 months during acute EBV infection adaptive features have still to be investigated.

In contrast to direct immune control of lytic EBV replication by early-differentiated NK cells (51), the role of terminally differentiated NK cells is less clear during HCMV infection. Only for decidual NKG2C-positive NK cells it was shown that they directly kill HCMV-infected autologous decidual fibroblasts in an HLA-E dependent fashion (72). Most studies, however, implicate the NKG2C-positive NK cell subset that expands during HCMV infection in mediating superior antibody-dependent cellular cytotoxicity (ADCC) against antibody opsonized HCMV-infected macrophages or fibroblasts (73, 74). In these studies, both IFN-γ production and degranulation of NKG2C-positive NK cells of HCMV-infected donors were superior upon opsonized target recognition compared to NKG2C-negative NK cell populations. These superior effector functions most likely result from epigenetic modifications, as has been shown for the IFN-γ gene locus in NKG2C-positive NK cells of HCMV infected individuals (75–78). Thus, early-differentiated NK cells that expand during EBV infection might directly recognize lytically EBV replicating targets, while the terminally differentiated NK cells in HCMV-infected individuals mainly promote ADCC.

## Author Contributions

All authors listed have made substantial, direct, and intellectual contribution to the work and approved it for publication.

## Conflict of Interest Statement

The authors declare that the research was conducted in the absence of any commercial or financial relationships that could be construed as a potential conflict of interest.
